# Supplementary material for: Bacterial viruses enable their host to acquire antibiotic resistance genes from neighbouring cells
Source: Nat Commun. 2016 Nov 7;7:13333. doi: 10.1038/ncomms13333 (PMC5103068; doi:10.1038/ncomms13333)
Supplement: Supplementary Information — Supplementary Figures 1-7, Supplementary Tables 1-4 and Supplementary References. [file ncomms13333-s1.pdf]

Supplementary Figure 1

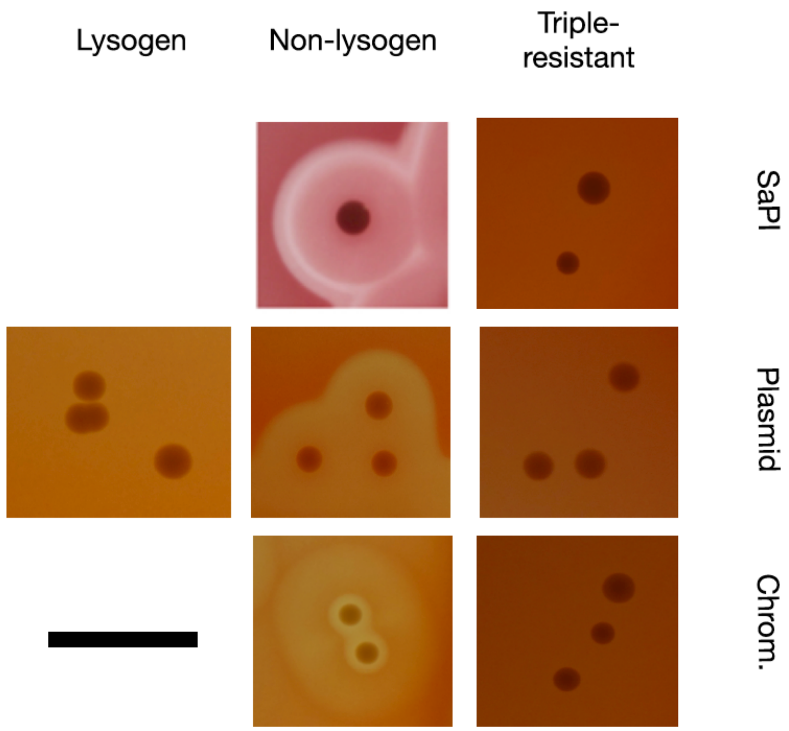

Supplementary Figure 1. Hemolysis phenotype of parental strains 8325-SR (lysogen), 8325-4 (non-lysogen) carrying SaPI, plasmid and chromosomal markers and triple resistant colonies in the 8325-SR co-cultures from fig. 1a. Scale bar indicates 10 mm.

Supplementary Figure 2

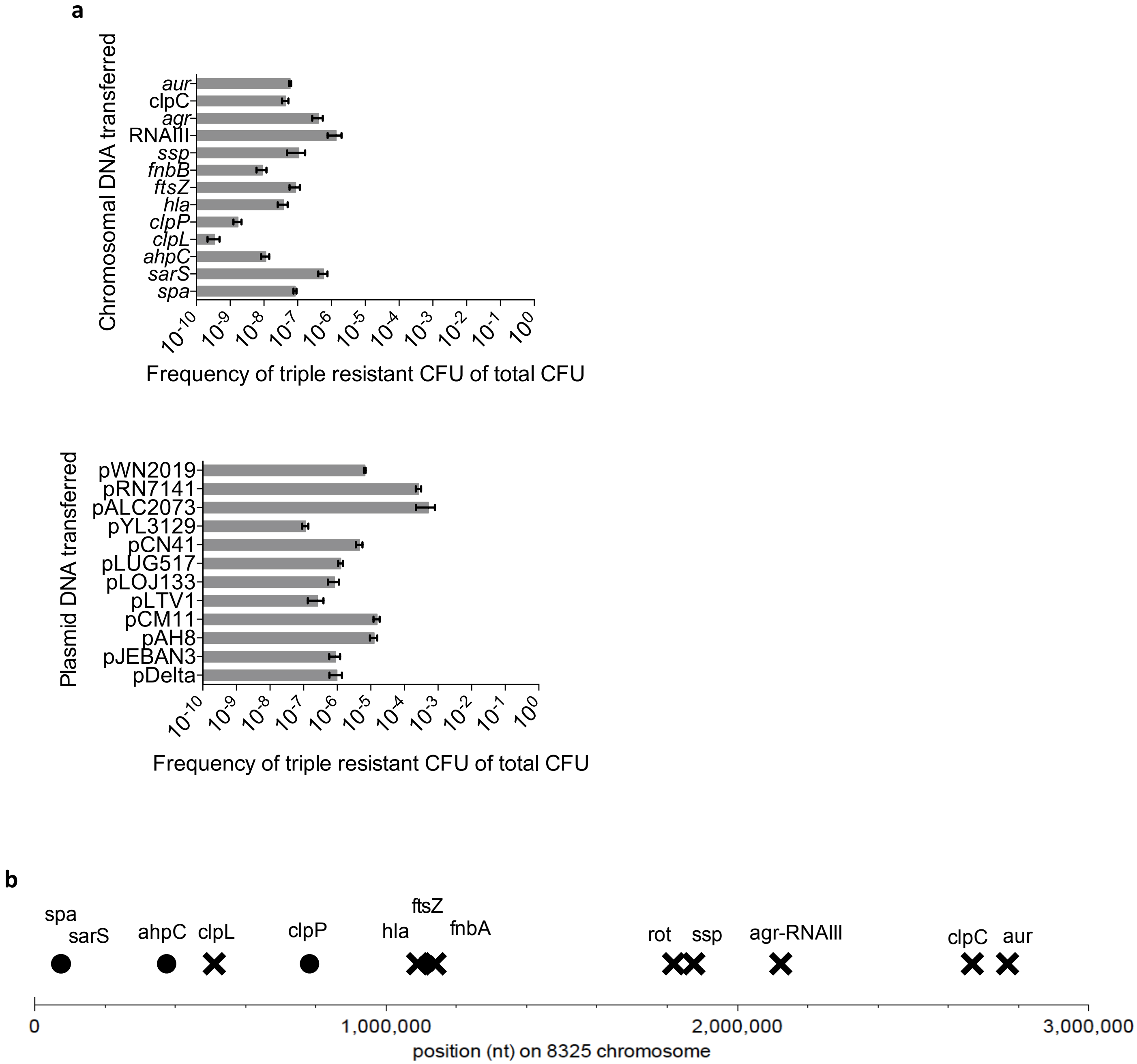

Supplementary Figure 2. Chromosomal and plasmid DNA transferred from phage-susceptible non-lysogenic to lysogenic populations. (a) Frequency of triple resistant cells after 1:1 ratio co-culture of 8325-SR and a susceptible strain harbouring resistance markers at various chromosomal positions (upper panel) or at plasmids (lower panel). **(b)** The position of the chromosomal resistance markers on the NCTC 8325-4 genome used in the transfer experiments shown in (a). The strains carrying a resistance marker at the position marked by “X” were genome sequenced and analysed for details of the DNA transfer. Error bars indicate standard deviation. n = 3.

Supplementary Figure 3

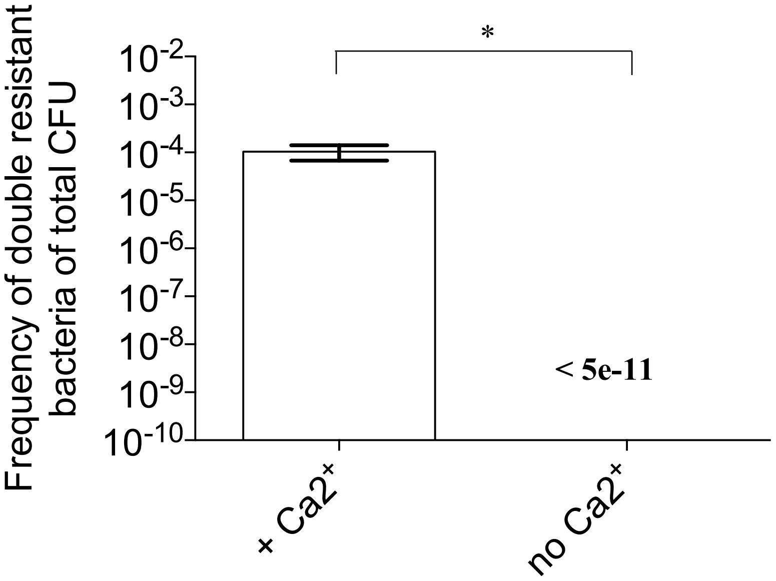

Supplementary Figure 3. Frequencies of double-resistant cells relative to total CFU were determined in co-cultures of 8325-S and 8325-4 plasmid with and without addition of 10 mM $CaCl_2$. *: $p < 0.05$ applying t-test.

Supplementary Figure 4

a

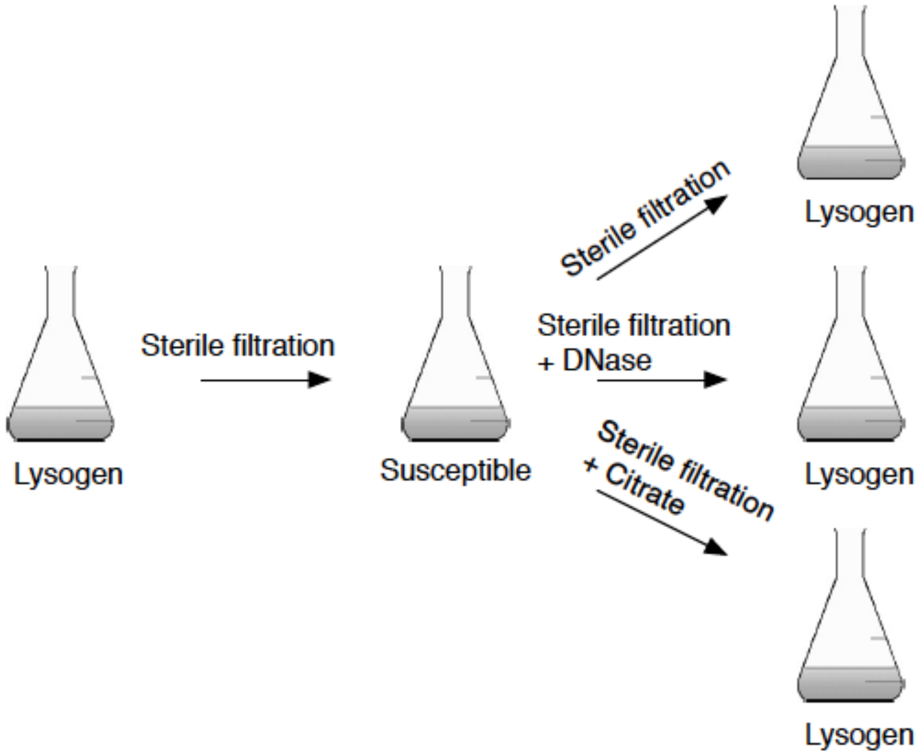

b

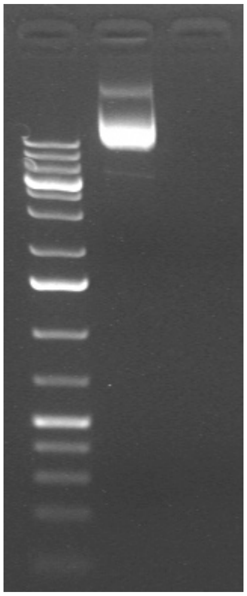

c

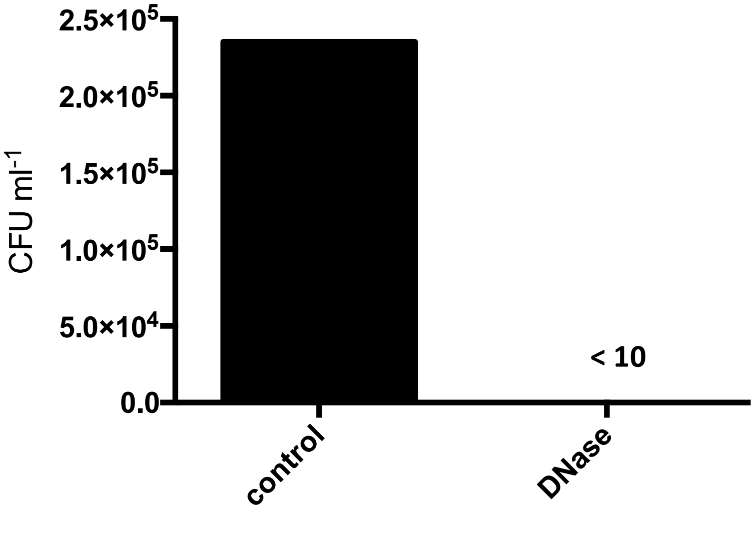

Supplementary Figure 4. Experimental setup and DNase control. (a) Step-wise methodology used to investigate whether DNA transfer was dependent on cell-cell contact or inhibited by exposure to DNase or citrate. Briefly, 8325-S was grown to mid-exponential phase during which phages were spontaneously released to the supernatant. Cell-free supernatant from this culture was used to infect 8325-4 SaPI and phages were allowed to propagate for 2 hours before harvesting the supernatant. Cell-free supernatant

from this culture was then used to infect lysogenic 8325-S with or without added DNaseI or citrate. After overnight incubation total CFU and CFU of double resistant mutants were determined. **(b)** pRMC2 plasmid DNA before (lane 2) and after (lane 3) DNaseI treatment. Lane 1 is 1kb plus DNA ladder (Fermentas). **(c)** CFU of *E. coli* DH5α cells electroporated with pRMC2 plasmid DNA after DNaseI treatment (DNase) or not exposed to DNase (control).

Supplementary Figure 5

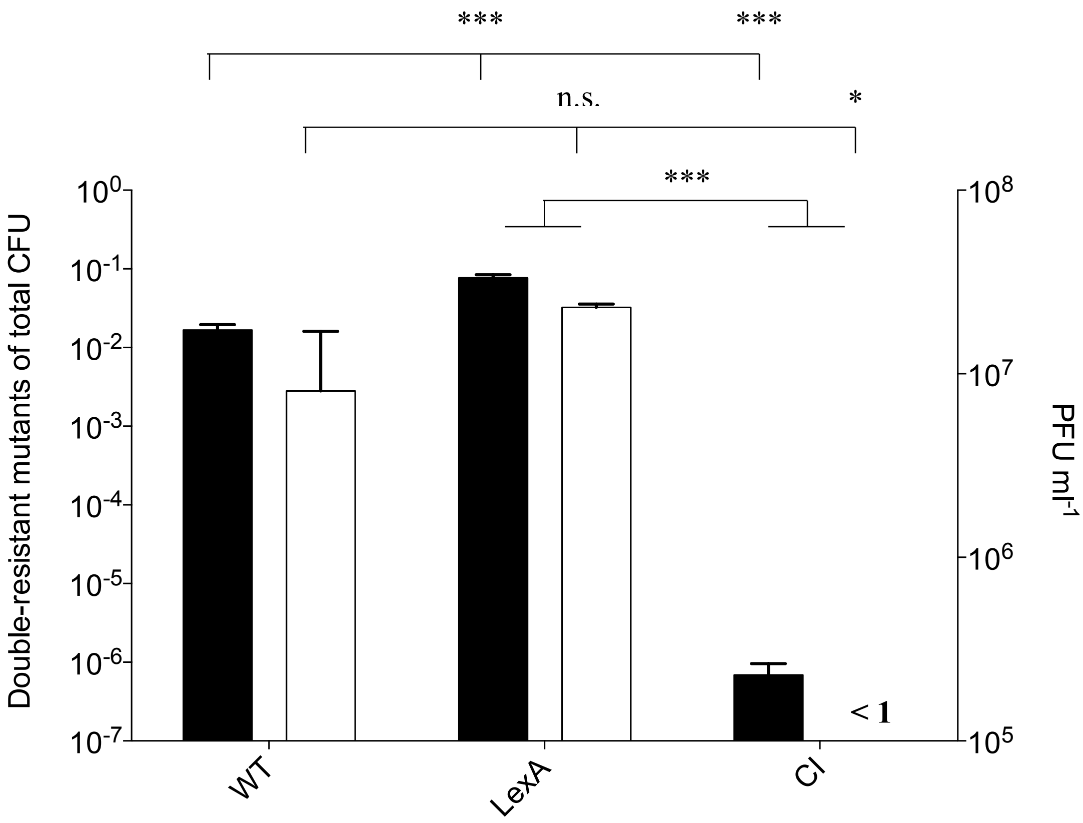

Supplementary Figure 5. Spontaneous phage induction and transfer of DNA is not dependent on SOS DNA damage response. Frequency of double resistant cells per total CFU (filled bars) and free phage titers (open bars) in the supernatant of co-cultures of 8325-4 SaPI with either lysogenic WT 8325-S; a lysogenic LexA mutant not capable of inducing SOS-response or a ϕ11 lysogen expressing a non-inducible CI phage repressor (CI). All lysogens were resistant to streptomycin. In all strains a basic level of *recA* expression was observed. Error bars = s.d., n=3. ***:$p<0.001$, *:$p<0.05$, n.s.: not significant when applying t-test.

Supplementary Figure 6

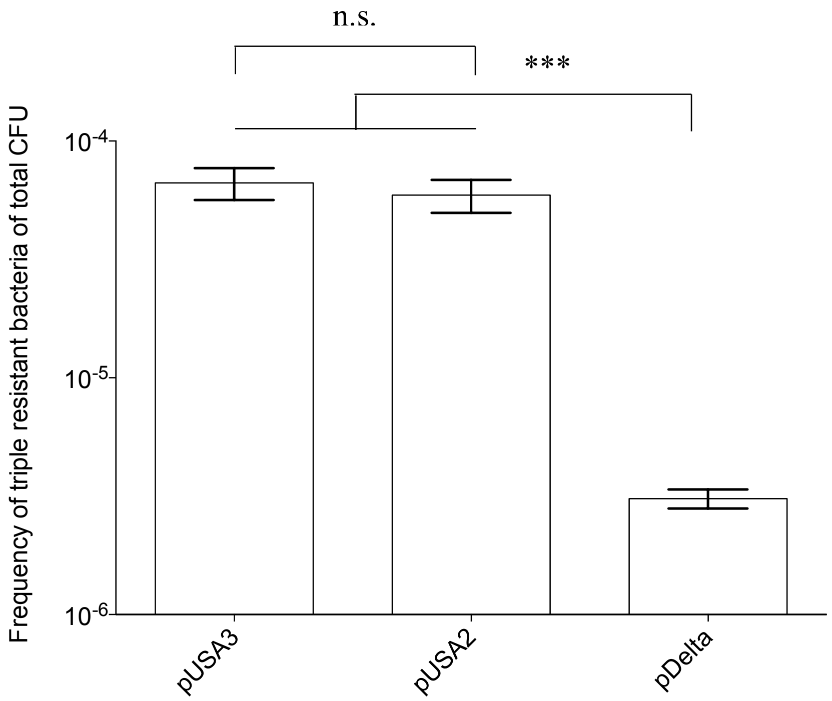

Supplementary Figure 6. Auto-transduction of DNA acquired from non-ϕ11 lysogenic strains. Auto-transduction frequencies in co-cultures of 8325-SR and USA300 carrying plasmids pUSA2 (*tetK*) and pUSA3 (*ermC*) or Newman carrying plasmid pDelta (*tetM*). Error bars = s.d., n=3. ***$p<0.001$, n.s.: not significant when applying t-test.

Supplementary Figure 7

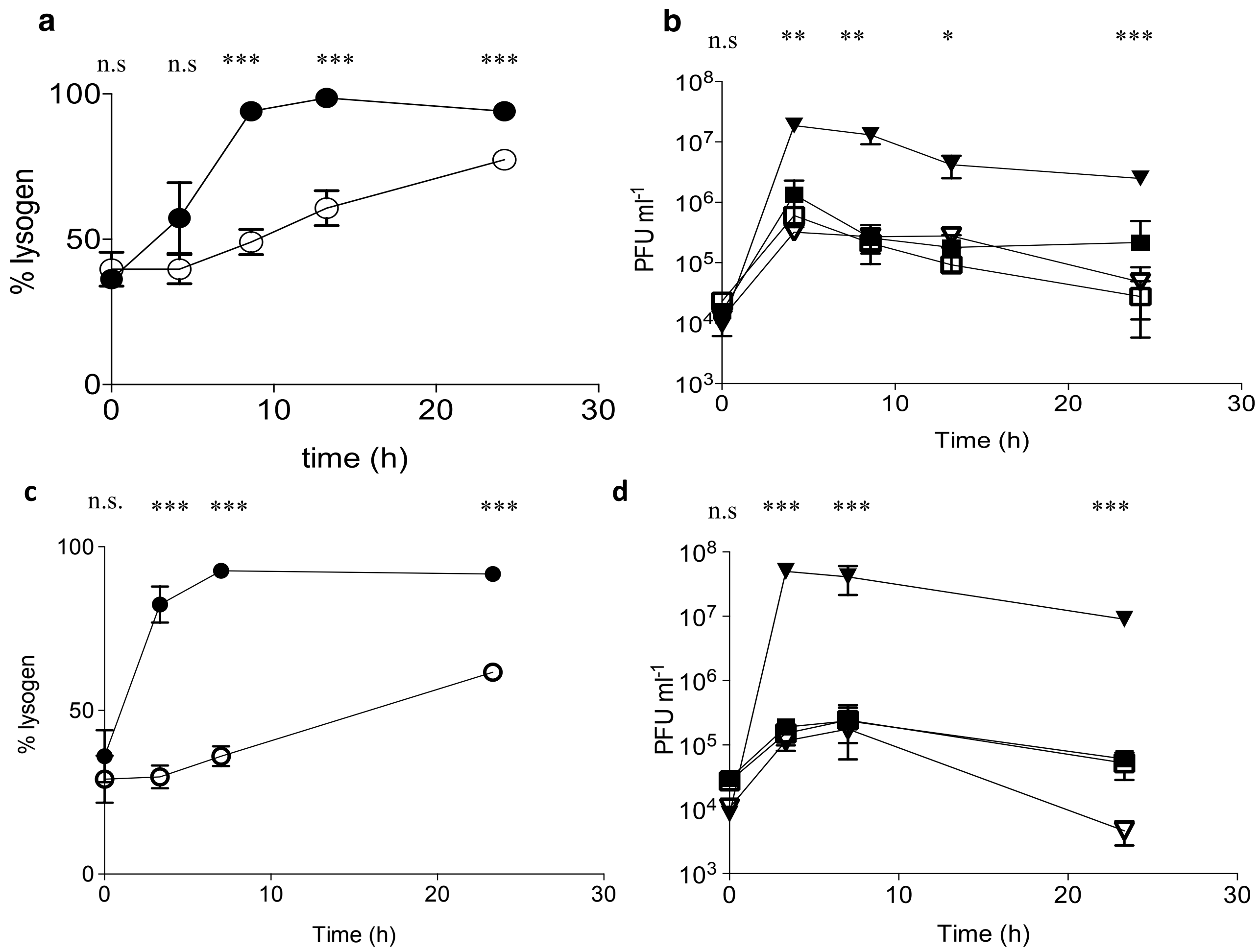

Supplementary Figure 7. Interference competition by phage release. (a,c) Competition measured as percentage lysogen of the total CFU over time obtained in 1:1 co-cultures of lysogen 8325-SR and phage susceptible 8325-4 chrom. **(a)** or USA300 **(c)** with (open symbols) and without (filled symbols) added citrate. (**b,d)** Free phage titers in the supernatant of mono-cultures of 8325-SR (square symbols) or 1:1 mixed cultures (triangle symbols) with 8325-4 chrom. **(b)** or USA300 **(d)** with (open symbols) and without (filled symbols) added citrate. When incubated in monocultures, exponential growth rates of 8325-SR were significantly lower than 8325-4 chrom. ($p<0.05$) and USA300 ($p<0.05$), See supplementary table 4. Error bars = s.d., n=3. ***:$p<0.001$, **:$p<0.01$, *:$p<0.05$, n.s.: not significant. Statistics calculated for mixed cultures with and without added citrate applying t-test.

Supplementary Table 1. Efficiency of plaquing (EOP) when lysates from 8325, Newman and USA300 with known number of phage particles were used to infect the same strains or the phage-cured 8325-derivative RN4220.

		Lysate from strain		
		8325	Newman	USA300
	RN4220	6.2e-2 ± 2.7e-3	6.5e-2 ± 1.0e-2	< 3.5e-7
EOP on strain	8325	< 2.3e-9	< 4.6e-8	< 3.5e-7
	Newman	7.6e-9 ± 1.3e-8	< 4.6e-8	< 3.5e-7
	USA300	1.7e-2 ± 3.0e-3	< 4.6e-8	< 3.5e-7

Supplementary Table 2. *S. aureus* strains used in this study.

Strain name	Relevant characteristics	Reference
8325-S	Streptomycin resistant derivative of 8325 (RN1) carrying prophages ϕ11, ϕ12 and ϕ13	This study
8325-SR	Rifampicin resistant derivative of 8325-S	This study
8325-4 SaPI	RN450 containing SaPIbov1 fused tetracycline resistance marker (tst::*tetM*), transduced from JP47	This study
8325-4 plasmid	pRMC2, non-conjugative plasmid (*cat*)	1
8325-4 chrom	WA525 = 8325-4, rot::ermB	2
RN451	Derivative of 8325 only carrying ϕ11	3
JH951	Streptomycin resistant derivative of RN451	This study
JH944	Streptomycin and rifampicin resistant derivative of RN451	This study
JP1622	RN451, ϕ11 CI G133E (non-inducible CI repressor)	4
JP47	RN451 (SaPIbov1 *tst*::*tet*M)	4
JH982	Streptomycin resistant derivative of JP1622	This study
CH4030	ϕ11, packing deficient *terL* mutant	5
JH950	Streptomycin resistant derivative of CH4030	This study
RN450	8325-4 = phage cured version of 8325	3
JH977	Streptomycin resistant derivative of RN450	This study
JH978	Streptomycin and rifampicin resistant derivative of RN450	This study
JP1879	RN451, lexA (G94E)	6
JH983	Streptomycin resistant derivative of JP1879	This study
USA300	CA-MRSA, pUSA2 (tetK), pUSA3 (ermC), prophage ϕSA3usa	7
Newman	Strain Newman, 3 active prophages, pTX plasmid (tetM)	8
DU5882	8325-4, fnbAB::ermC	9
HI2207	8325-4, clpL::erm	10
AK1	8325-4, aur::ermB	11
AK2	8325-4, ssp::ermB	11
DU1090	8325-4, hla::erm	12
HI2343	8325-4, clpC::erm	10
PC203	8325-4, Spa promoter::ermB	13
SaΔclpP	8325-4, clpP::erm	14
SH101F7	8325-4, RNAIII promoter::ermB	15
ALC1927	8325-4, pSarA::lacZ (erythromycin resistant)	16
RNpFtsz-1	RN4220, inducible FtsZ::ermB	17
KT202	8325-4, agr::tetM	18
KC043	SH1000, ahpC::tet	19
HI2371	RN4220, pYL3129 (cam)	20
ALC2158	8325-4, pALC2073 (cam)	21
RN9723	RN6734 (8325-4), pJW7141 (cam)	22

RN8019	8325-4, pRN6832 (cam)	23
MTC459	8325-4, pΔsosA (erm)	24
8325-4-YFP	8325-4, pJEBAN3 (erm)	25
AH492	RN4220, pAH8 (erm)	26
AH1331	RN4220, pCM11 (erm)	27
HI2406	RN4220, pLTV1 (erm)	28
LJ98	8325-4, pLOJ133 (erm)	29
LUG790	RN6390, pLUG517 (erm)	30
RN9596	RN4220, pCN41 (erm)	31
MTC174	8325-4, recA promoter::lacZ, tetracycline resistant	32
JH984	8325-SR, recA::lacZ from MTC174 (strep^R, rif^R, tet^R)	This study
JH986	JP1622, recA::lacZ from MTC174 (tet^R)	This study
JH985	RN451, recA::lacZ from MTC174 (tet^R)	This study

Supplementary Table 3. Spontaneous antibiotic resistance mutations.

	Frequency of spontaneous resistance to:					
	Streptomycin	Rifampicin	Streptomycin and rifampicin	Tetra-cycline	Chloram-phenicol	Erythro-mycin
8325-S	n.a.	<8.2e-10	n.a.	<8.2e-10	5.9e-9 ± 6.1e-11	<8.2e-10
8325-SR	n.a.	n.a.	n.a.	<3.1e-10	<3.1e-10	<3.1e-10
8325-4 SaPI	2.5e-7 ± 2.5e-7	7.3e-8 ± 6.3e-8	<2.3e-10	n.a.	1.6e-9 ± 2.8e-9	<2.3e-10
8325-4 plasmid	2.9e-8 ± 5.0e-8	9.6e-7 ± 7.9e-7	<4.5e-9	<4.5e-9	n.a.	<1.7e-9
8325-4 chrom.	3.0e-7 ± 1.6e-8	2.5e-7 ± 1.6e-7	<3.5e-10	<3.5e-10	1.5e-8 ± 4.8e-9	n.a.

n.a. = not applicable

Supplementary Table 4. Average specific growth rates of relevant strains

Strain	Growth rate* h^{-1}	Standard.dev
8325-S	0,62	0,02
8325-SR	0,66	0,07
8325-4 SaPI	0,60	0,02
8325-4 plasmid	0,65	0,05
8325-4 chrom.	0,75	0,01
JH951	0,60	0,01
JH982	0,70	0,05
JH950	0,57	0,03
USA300	0,76	0,03
Newman	0,73	0,01

*) n = 5

Supplementary References

1 Corrigan, R. M. & Foster, T. J. An improved tetracycline-inducible expression vector for *Staphylococcus aureus*. *Plasmid* **61**, 126-129 (2009).

2 Oscarsson, J., Harlos, C. & Arvidson, S. Regulatory role of proteins binding to the *spa* (protein A) and *sarS* (staphylococcal accessory regulator) promoter regions in *Staphylococcus aureus* NTCC 8325-4. *Int. J. Med. Microbiol.* **295**, 253-266 (2005).

3 Novick, R. Properties of a cryptic high-frequency transducing phage in *Staphylococcus aureus*. *Virology* **33**, 155-166 (1967).

4 Ubeda, C. *et al.* Antibiotic-induced SOS response promotes horizontal dissemination of pathogenicity island-encoded virulence factors in staphylococci. *Mol. Microbiol.* **56**, 836-844 (2005).

5 Quiles-Puchalt, N. *et al.* Staphylococcal pathogenicity island DNA packaging system involving *cos*-site packaging and phage-encoded HNH endonucleases. *Proc. Natl. Acad. Sci. USA.* **111**, 6016-6021 (2014).

6 Ubeda, C. *et al.* SaPI operon I is required for SaPI packaging and is controlled by LexA. *Mol. Microbiol.* **65**, 41-50 (2007).

7 Diep, B. A. *et al.* Complete genome sequence of USA300, an epidemic clone of community-acquired meticillin-resistant *Staphylococcus aureus*. *Lancet* **367**, 731-739 (2006).

8 Paulander, W. *et al.* Antibiotic-mediated selection of quorum-sensing-negative *Staphylococcus aureus*. *MBio.* **3,** e00459-12 (2013).

9 Greene, C. *et al.* Adhesion properties of mutants of *Staphylococcus aureus* defective in fibronectin-binding proteins and studies on the expression of fnb genes. *Mol. Microbiol.* **17**, 1143-1152 (1995).

10 Frees, D. *et al.* Clp ATPases are required for stress tolerance, intracellular replication and biofilm formation in *Staphylococcus aureus*. *Mol. Microbiol.* **54**, 1445-1462 (2004).

11 Karlsson, A., Saravia-Otten, P., Tegmark, K., Morfeldt, E. & Arvidson, S. Decreased amounts of cell wall-associated protein A and fibronectin-binding proteins in *Staphylococcus aureus sarA* mutants due to up-regulation of extracellular proteases. *Infect. Immun.* **69**, 4742-4748 (2001).

12 Bayer, A. S. *et al.* Hyperproduction of alpha-toxin by *Staphylococcus aureus* results in paradoxically reduced virulence in experimental endocarditis: a host defense role for platelet microbicidal proteins. *Infect. Immun.* **65**, 4652-4660 (1997).

13 Chan, P. F. & Foster, S. J. The role of environmental factors in the regulation of virulence-determinant expression in *Staphylococcus aureus* 8325-4. *Microbiol.* **144**, 2469-2479 (1998).

14 Frees, D., Qazi, S. N., Hill, P. J. & Ingmer, H. Alternative roles of ClpX and ClpP in *Staphylococcus aureus* stress tolerance and virulence. *Mol. Microbiol.* **48**, 1565-1578 (2003).

15 Horsburgh, M. J. *et al.* sigmaB modulates virulence determinant expression and stress resistance: characterization of a functional rsbU strain derived from *Staphylococcus aureus* 8325-4. *J. Bacteriol.* **184**, 5457-5467 (2002).
16 Cheung, A. L., Schmidt, K., Bateman, B. & Manna, A. C. SarS, a SarA homolog repressible by *agr*, is an activator of protein A synthesis in *Staphylococcus aureus*. *Infect. Immun.* **69**, 2448-2455 (2001).
17 Pinho, M. G. & Errington, J. Dispersed mode of *Staphylococcus aureus* cell wall synthesis in the absence of the division machinery. *Mol. Microbiol.* **50**, 871-881 (2003).
18 Tegmark, K., Karlsson, A. & Arvidson, S. Identification and characterization of SarH1, a new global regulator of virulence gene expression in *Staphylococcus aureus*. *Mol. Microbiol.* **37**, 398-409 (2000).
19 Cosgrove, K. *et al.* Catalase (KatA) and alkyl hydroperoxide reductase (AhpC) have compensatory roles in peroxide stress resistance and are required for survival, persistence, and nasal colonization in *Staphylococcus aureus*. *J. Bacteriol.* **189** (2007).
20 Lim, Y., Jana, M., Luong, T. T. & Lee, C. Y. Control of glucose- and NaCl-induced biofilm formation by rbf in *Staphylococcus aureus*. *J. Bacteriol.* **186**, 722-729 (2004).
21 Bateman, B. T., Donegan, N. P., Jarry, T. M., Palma, M. & Cheung, A. L. Evaluation of a tetracycline-inducible promoter in *Staphylococcus aureus in vitro* and *in vivo* and its application in demonstrating the role of *sigB* in microcolony formation. *Infect. Immun.* **69**, 7851-7857 (2001).
22 Wright, J. S., III, Jin, R. & Novick, R. P. Transient interference with staphylococcal quorum sensing blocks abscess formation. *Proc. Natl. Acad. Sci. USA* **102**, 1691-1696 (2005).
23 Kornblum, J. K., B.; Projan, S. J.; Ross, H.; Novick, R. P. in *Molecular biology of the staphylococci* (ed R. P. Novick) p.373-402 (VCH Publishers, 1990).
24 Cohn, M. T., Kjelgaard, P., Frees, D., Penades, J. R. & Ingmer, H. Clp-dependent proteolysis of the LexA N-terminal domain in *Staphylococcus aureus. Microbiol.* **157**, 677-684 (2011).
25 Haaber, J., Cohn, M. T., Frees, D., Andersen, T. J. & Ingmer, H. Planktonic aggregates of *Staphylococcus aureus* protect against common antibiotics. *PLoS ONE.* **7**, e41075 (2012).
26 Malone, C. L. *et al.* Fluorescent reporters for *Staphylococcus aureus*. *J. Microbiol. Methods.* **77**, 251-260 (2009).
27 Lauderdale, K. J., Malone, C. L., Boles, B. R., Morcuende, J. & Horswill, A. R. Biofilm dispersal of community-associated methicillin-resistant *Staphylococcus aureus* on orthopedic implant material. *J. Orthop. Res.* **28**, 55-61 (2010).
28 Youngman, P., Poth, B., Green K., York, G, Olmedo, G, Smith, K. in *Regulation of procaryotic development* (ed R. A. Smith, Slepecky, R. A., Setlow, P) (American Society for Microbiology,, 1989).
29 Jelsbak, L. *et al.* The chaperone ClpX stimulates expression of *Staphylococcus aureus* protein A by Rot dependent and independent pathways. *PLoS ONE.* **5**, e12752 (2010).

30 Huntzinger, E. *et al.* *Staphylococcus aureus* RNAIII and the endoribonuclease III coordinately regulate *spa* gene expression. *EMBO J.* **24**, 824-835, doi:10.1038/sj.emboj.7600572 (2005).
31 Charpentier, E. *et al.* Novel cassette-based shuttle vector system for gram-positive bacteria. *Appl. Environ. Microbiol.* **70**, 6076-6085 (2004).
32 Gottschalk, S. *et al.* The amphibian antimicrobial peptide fallaxin analogue, FL9, affects virulence gene expression and DNA replication in *Staphylococcus aureus*. *J. Med. Microbiol* **64,** 1504-1513 (2015).
